# Nutrition in Post-Stroke Subjects during Rehabilitation

**DOI:** 10.3390/nu15092056

**Published:** 2023-04-25

**Authors:** Mariacristina Siotto, Irene Aprile

**Affiliations:** IRCCS Fondazione Don Carlo Gnocchi, 50143 Florence, Italy; iaprile@dongnocchi.it

Correct and appropriate nutrition after a stroke insult appears to exert an essential influence on, and play a key role in, the recovery of patients. The clinical picture after stroke can be heterogeneous, and its evolution and response to the rehabilitation can differ significantly despite the similar clinical status of patients at the onset. Individual factors can modulate the clinical picture and response to the rehabilitation treatment. There exist very few studies regarding the importance of nutrition after a stroke insult, even though the high prevalence of malnutrition among these patients can have significant impacts on their physical and cognitive functions. The aim of this Special Issue is to examine the current state of diet and nutrition research on post-stroke subjects admitted to rehabilitation centers. Of the six manuscripts published in this issue, three are original articles and three are narrative reviews.

Two of three studies analyzed albumin, prealbumin, and transferrin as nutritional biomarkers in post-stroke patients. Park et al. studied these nutritional biomarkers in association with heart rate variability [1], while Kim et al. examined them as predictors of dysphonia severity [2]. 

Since malnutrition and autonomic dysfunction are associated with poor outcomes, mortality, and psychological problems after stroke, the study of Park and colleagues examined heart rate variability parameters among 426 subacute post-stroke patients, who underwent 24 h ambulatory Holter electrocardiography [1]. The heart rate variability parameters were significantly lower in the groups with nutritional biomarker deficiencies, and there was a significant association between the heart rate variability parameters and nutritional biomarkers. Furthermore, serum nutritional biomarkers were associated with autonomic nervous system function, as measured by heart rate variability, and it was found that their deficiency may be a predictor of the severity of autonomic nervous system dysfunction in stroke patients [1].

Together with malnutrition, dysphonia is a major problem in patients who have experienced an ischemic stroke. The study conducted by Kim and colleagues [2] found that serum levels of transferrin, albumin, and prealbumin were significantly correlated with the dysphonia severity index and maximum phonation time levels. In a multiple regression analysis, prealbumin and transferrin were found to be significant predictors of the dysphonia severity index, whereas prealbumin alone was a significant predictor of the maximum phonation time. These results may provide objective evidence showing that nutritional biomarkers influence the severity of dysphonia.

The third study investigated the relationship between nutritional status, food consumption, and sarcopenia in post-stroke patients during a six-week rehabilitation treatment [3]. Siotto and colleagues found that the patients affected by sarcopeniadiagnosed according to the EWGSOP2 guidelines, had a worse functional recovery and nutritional status. In fact, the MNA-SF^®^ score on admission was lower, together with a lower BMI and a lower Geriatric Nutritional Risk Index both on admission and after six weeks of the rehabilitation program. In addition, the sarcopenic patients discarded one-third of their daily meals on average throughout the duration of the study. This study suggests that a correct diagnosis of sarcopenia is necessary for post-stroke patients in order to design an individually targeted physical and nutritional intervention to improve the clinical outcomes of these patients [3].

The reviews included in this Special Issue all conclude that studies on nutrition in post-stroke patients during rehabilitation are too scarce and that this field deserves more attention from the scientific community. 

A proper/improper nutritional status can influence rehabilitation after a stroke, as highlighted in the review by Ciancarelli and colleagues [4]. The authors emphasized the influences of oxidative stress and inflammation on neuro-rehabilitative outcomes. After an ischemic cascade attack, neuronal death and brain infarction enhance the production of free radicals, which, in turn, may promote the expansion of the ischemic lesion, blood–brain barrier dysfunction, and systemic inflammatory response syndrome, which negatively affect the stroke outcome. Thus, the brain recovery process requires specific nutritional components with antioxidant and anti-inflammatory effects. Unfortunately, the results regarding the positive effects of antioxidant and anti-inflammatory food components or foods, with respect to the rehabilitation outcomes of malnourished stroke patients, are based on small and non-uniformly selected samples. Moreover, since different rehabilitation outcome were analyzed to assess patients’ recovery, the results are not entirely conclusive. Other criticisms include the heterogeneous assessment of malnutrition at various centers and the absence of consistent data on the effectiveness of supplementation after the reintegration of subjects into society and daily life [4]. 

A similar conclusion was reached in the narrative review of Marek and colleagues, in which the role of vitamin D in stroke prevention and its supplementation in post-stroke patients during rehabilitation were analyzed [5]. Although vitamin D has been shown to contribute to improved rehabilitation outcomes in stroke patients, studies testing the efficacy of vitamin D supplementation in post-stroke patients face many limitations that affect the results. Moreover, these studies are very scarce, and researchers have stated the need to expand well-designed studies in order to test the efficacy of vitamin D supplementation. Ideally, a randomized controlled clinical trial with a large sample and a follow-up of at least five years should be attempted.

Finally, the review of Di Vincenzo and colleagues [6] highlights an important problem which summarizes the aim of this Special Issue: malnutrition is a widespread problem in post-stroke patients undergoing rehabilitation, and it can be assumed that little effort has been made to identify a well-defined nutritional care protocol. Nutritional risk assessments in both the acute and subacute phases of stroke are differentiated, and few attempts have been made to compare the different tools/procedures in terms of usefulness and reliability. Moreover, we are still far from identifying the most appropriate and well-defined nutritional screening procedures, which should be quick, simple, cost-effective, and reliable in clinical settings [6]. 

The set of studies collected in this Special Issue suggests the urgent need for future research that better defines how to assess nutritional risk during the hospitalization of patients after a stroke insult and during their stay in rehabilitation centers. In order to enable the best possible recovery, the healthcare system should pay more attention to identify the best practices for malnutrition prevention and/or treatment using diet or supplementation in the management of post-stroke patients.
